# Dual Left Anterior Descending, Interarterial Principal LAD, and Anomalous Circumflex, Presenting With Acute Coronary Syndrome

**DOI:** 10.1016/j.jscai.2022.100402

**Published:** 2022-07-09

**Authors:** Kristen N. Brown, Calvin Craig, Andrew M. Goldsweig, Gregory Pavlides

**Affiliations:** Division of Cardiovascular Diseases, University of Nebraska Medical Center, Omaha, Nebraska

**Keywords:** computed tomography, coronary artery anomaly, percutaneous coronary intervention

Coronary artery anomalies are rare, but even rarer are the constellations of coronary anomalies. This article describes a very rare constellation of anomalous coronary arteries identified in the context of a non−ST-elevation myocardial infarction (NSTEMI). In addition to describing this challenging case, this article provides tips and tricks for operators on catheter selection to optimize the engagement of these atypical coronary arteries and highlights the high-risk features of coronary anomalies based on the arterial course, ostium shape, and vessel size.

## Case Description

**Figure. 1:**
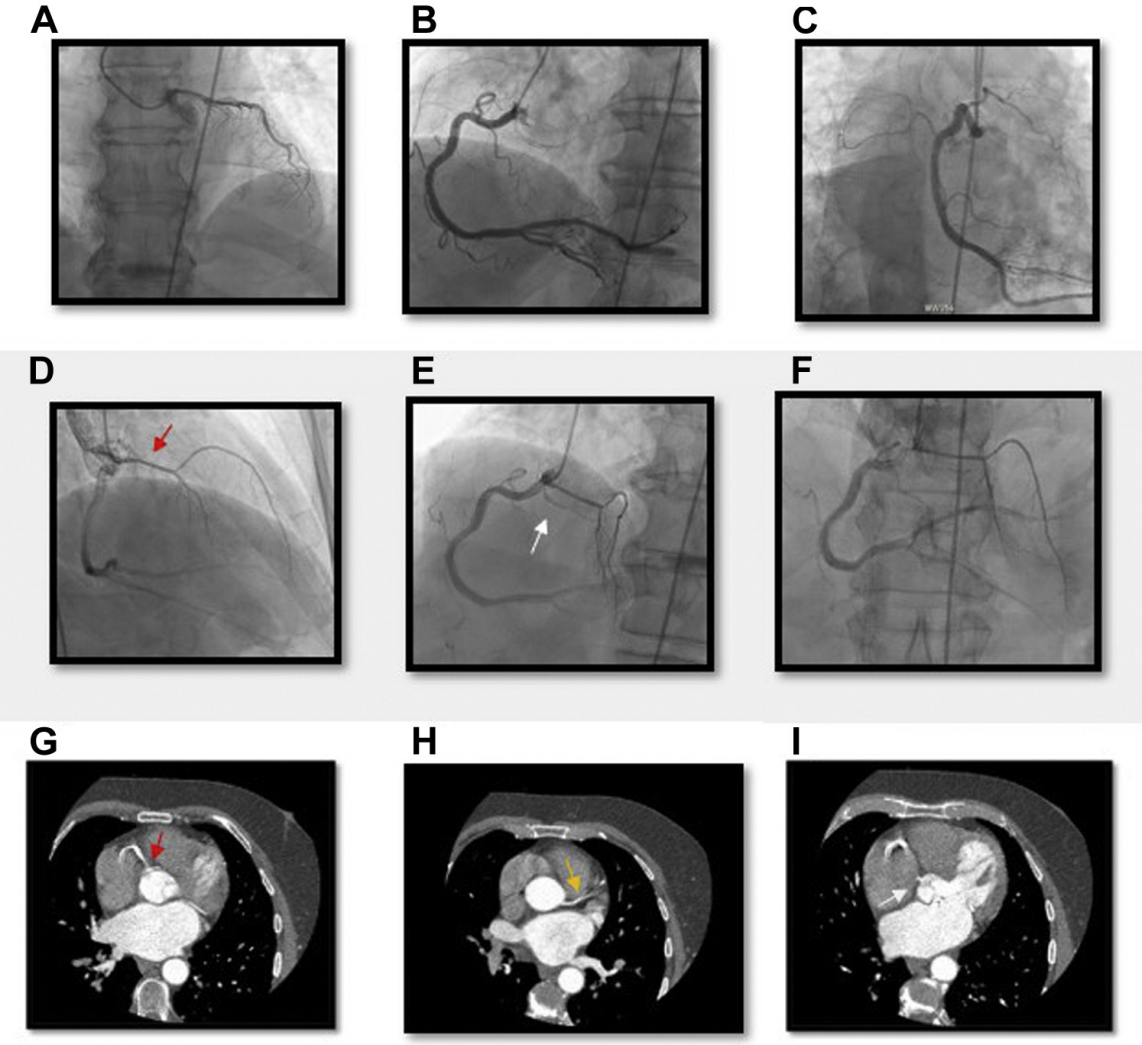
**Coronary angiography and computed tomography angiography demonstrating multiple coronary anomalies.** (**A**) Secondary LAD artery from the left coronary cusp using the JL3.5 in the RAO view. (**B**) RCA selectively engaged in the LAO view. (**C**) RCA selectively engaged in the RAO caudal view. (**D**) RCA with the principal LAD artery (red arrow) in the RAO cradial view. (**E**) RCA angiogram in the LAO cranial view revealing all 3 right-sided vessels including the LCx artery (white arrow). (**F**) RCA angiogram revealing the principal LAD artery branching from the RCA in the AP cranial view. (**G**) Principal LAD artery (red arrow) branching from the RCA. (**H**) Secondary LAD artery (yellow arrow) branching from the left coronary cusp. (**I**) LCx artery with a separate ostium branching from the RCC. AP, anteroposterior; LAD, left anterior descending; LAO, left anterior oblique; LCx, left circumflex; RAO, right anterior oblique; RCA, right coronary artery; RCC, right coronary cusp.

A 73-year-old man presented to the emergency department with chest pain and shortness of breath. His past medical history included coronary artery disease status after remote stenting of the right coronary artery (RCA), hypertension, hyperlipidemia, atrial fibrillation, nonsustained ventricular tachycardia, and chronic heart failure with preserved ejection fraction. An initial electrocardiogram showed rate-controlled atrial fibrillation with no significant ST-segment or T-wave changes. Troponin I level increased from 0.04 to 14.29 ​ng/mL. Given his NSTEMI, coronary angiography was performed, identifying the anomalous coronary anatomy (Figure 1): a dual left anterior descending (LAD) arterial system with the principal LAD artery arising from the RCA and an anomalous left circumflex (LCx) culprit artery arising from the right coronary cusp (RCC). The anomalous LAD artery was engaged with a Judkins right 4 (JR4) catheter (Cordis), whereas the anomalous LCx artery was cannulated with an Amplatz right 1 catheter (Boston Scientific). The NSTEMI culprit lesion was a ruptured atherosclerotic plaque in the proximal anomalous LCx artery. The LCx artery was treated with percutaneous coronary intervention with a 2.5 mm × 18 mm Onyx (Medtronic) drug-eluting stent. The patient remained hemodynamically stable throughout the procedure. The left ventricular end-diastolic pressure was 8 ​mm Hg. Following intervention, his anatomy was further characterized by computed tomography (CT) imaging, which revealed an interarterial course of the LAD artery inferior to the pulmonic valve (Figure 1). Given his age, he was managed medically and not referred for bypass grafting or unroofing of the LAD artery.

## Discussion

The origin of all 3 coronary arteries arising from the RCC is exceedingly rare. The true incidence of this coronary anomaly within the population is difficult to assess because sampling is limited to patients undergoing invasive angiography or noninvasive high-resolution cardiac imaging. To date, only a few cases have been described in the literature. This uncommon anomaly, combined with a dual LAD arterial system, makes the present case remarkable. This challenging anatomy made the diagnosis and management of acute myocardial infarction in the anomalous LCx artery particularly complex. The complexity of management was further compounded by the interarterial course of the principal LAD artery, later discovered on CT imaging.

The diagnosis of coronary anomalies can be challenging, even under stable conditions. The anomalies may be easily overlooked. Detection and characterization require experienced operators and a high index of suspicion.

Percutaneous coronary intervention in anomalous coronaries may also be arduous. Appropriate catheter selection is vital. For example, for an anomalous LCx artery originating from the RCC, the use of a right-sided catheter such as the Amplatz left or right, JR4, multipurpose (Asahi Intecc USA Inc), or hockey stick (Cordis) may be best.^1^ A preferred technique is to engage the RCA and rotate toward the ostium of the circumflex. For the LAD artery branching off the RCA, the JR4 ​or hockey stick would also be a reasonable catheter. Subsequently, engaging and wiring the RCA to anchor the catheter may allow the operator to retract the guide back to the anomalous LAD artery for the selective engagement of the LAD artery.

Anomalous LAD arteries may have retroaortic, prepulmonic, or interarterial courses, the last seen in this case. Patients with an interarterial course of the LAD artery may be referred for surgical intervention given the risk of sudden cardiac death from aortopulmonary compression and to alleviate anginal symptoms or syncope. In addition to using CT angiography,^2^ invasive angiography can identify an interarterial course in the right anterior oblique caudal view as a straight arterial course or with the use of a Swan-Ganz catheter to mark the pulmonary artery.^1^

Although most coronary anomalies are benign, guidelines recommend surgical intervention for interarterial or intermuscular malignant anomalous LAD artery courses if there is any evidence of ischemia.^3^ In the absence of ischemia or other concerning findings, the guidelines suggest that it may be reasonable to monitor these patients closely. The only exception here is the involvement of a left main branching off the right sinus with a concomitant interarterial course.^3^ Our patient, however, had lived with this anomaly for 7 ​decades without symptomatology; thus, the decision was to defer the surgical intervention. Additionally, more recent data suggest that surgical intervention carries substantial risks,^4^ further justifying the decision to take a less aggressive approach.
